# Functional brain networks related to individual differences in human intelligence at rest

**DOI:** 10.1038/srep32328

**Published:** 2016-08-26

**Authors:** Luke J. Hearne, Jason B. Mattingley, Luca Cocchi

**Affiliations:** 1 Queensland Brain Institute, The University of Queensland, Brisbane, Australia; 2 School of Psychology, The University of Queensland, Brisbane, Australia; 3 QIMR Berghofer Medical Research Institute, Brisbane, Australia

## Abstract

Intelligence is a fundamental ability that sets humans apart from other animal species. Despite its importance in defining human behaviour, the neural networks responsible for intelligence are not well understood. The dominant view from neuroimaging work suggests that intelligent performance on a range of tasks is underpinned by segregated interactions in a fronto-parietal network of brain regions. Here we asked whether fronto-parietal interactions associated with intelligence are ubiquitous, or emerge from more widespread associations in a task-free context. First we undertook an exploratory mapping of the existing literature on functional connectivity associated with intelligence. Next, to empirically test hypotheses derived from the exploratory mapping, we performed network analyses in a cohort of 317 unrelated participants from the Human Connectome Project. Our results revealed a novel contribution of across-network interactions between default-mode and fronto-parietal networks to individual differences in intelligence at rest. Specifically, we found that greater connectivity in the resting state was associated with higher intelligence scores. Our findings highlight the need to broaden the dominant fronto-parietal conceptualisation of intelligence to encompass more complex and context-specific network dynamics.

Human intelligence can be broadly defined as the capacity to understand complex ideas, adapt effectively to the environment and engage in complex reasoning^1^. Measures of intelligence can be related to performance on virtually any cognitive task, from sensory discrimination^2^ to challenging cognitive tasks such as the identification of patterns in the Raven’s Progressive Matrices test^3^. Importantly, scores on intelligence tests can accurately predict various life outcomes, including academic success^4^, job performance^5^, and adult morbidity and mortality^6^. Brain imaging studies have suggested that neural activity in frontal and parietal cortices during the execution of cognitive tasks is related to individual differences in intelligence^7^,^8^. These findings have been formalised in the influential Parieto-Frontal Integration Theory of intelligence (P-FIT)^9^, and have been proposed to extend to intrinsic networks of the brain^10^,^11^. By contrast, recent work investigating brain activity at rest (i.e., in the absence of any specific cognitive task) has suggested intelligence is underpinned by communication between widespread brain regions including, but not limited to, parieto-frontal areas^12^,^13^,^14^. Here we asked whether the P-FIT extends to intrinsic brain networks by undertaking an explorative summary of recent literature and conducting an empirical analysis using a dataset of 317 unrelated participants from the Human Connectome Project^15^.

Functional magnetic resonance imaging (fMRI) has been used to examine the relationship between individual differences in intelligence and brain activity during the engagement of cognitive abilities such as working memory^16^ and reasoning^17^,^18^. Typically, in these studies regions associated with intelligence are isolated by subtracting fMRI signals between two conditions with different ‘intelligence-loadings’ (e.g., easy versus difficult reasoning problems)^17^,^18^. Local changes in brain activity are then correlated with standard intelligence scores to identify regions that are related to individual differences in intelligence. Although this approach has been useful for identifying functionally segregated neural correlates of intelligence, it is insensitive to the integration of information processes across spatially and functionally segregated brain regions (e.g., functional connectivity).

Recent studies have investigated the relationship between individual intelligence scores and patterns of functional connectivity during resting state scans^10^,^14^,^19^. One hypothesis to emerge from this work is that resting state functional connectivity associated with intelligence should recapitulate the functional topology of frontal-parietal networks^10^. Attempts to test this prediction, however, have so far produced inconclusive findings. For example, individual differences in intelligence have been related to changes in resting state connectivity in neural networks broadly involved in self-referential mental activity (default-mode network), attentional control processes (dorsal attention network) and task-set maintenance (cingulo-opercular network)^12^,^20^,^21^,^22^,^23^,^24^.

Here we used convergent approaches to assess whether the P-FIT can be extended to task-free (resting state) contexts. We started by conducting an exploratory mapping of previous findings from studies that had investigated the relationship between resting state functional connectivity and measures of intelligence. Specifically, we mapped significant pairwise connections from four previous studies (see Table 1) into a validated topological characterisation of resting state brain networks^25^. We found that the previously reported functional connections associated with intelligence were not restricted to the fronto-parietal system (Fig. 1). We next tested this qualitative observation by mapping brain-intelligence relationships using a large and independent set of neuroimaging and behavioural data from the Human Connectome Project (HCP)^15^. Within the HCP data, general intelligence is defined as individual scores on a shortened version of the Raven’s Progressive Matrices and the Picture Vocabulary test. According to a context-invariant interpretation of the P-FIT^10^, intelligence should be related to connectivity within a fronto-parietal network as assessed during task performance and in the resting state. Conversely, absence of overlap between task and resting state networks would be more consistent with a context-specific neurophysiological model of intelligence.

## Results

### Exploratory mapping: resting state functional connectivity related to intelligence

**Figure 1:**
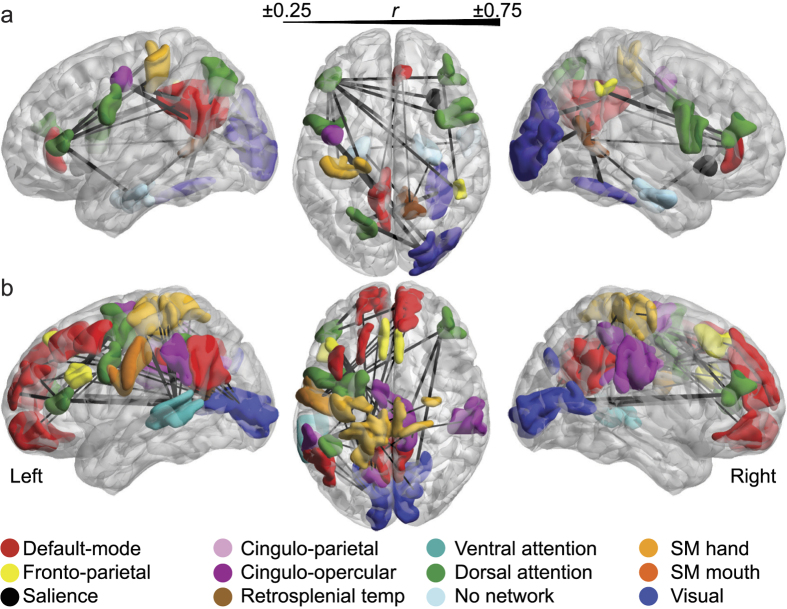
Pairwise functional connections associated with intelligence at rest from previous literature. (**a**) Connections in which higher functional connectivity was associated with higher intelligence. (**b**) Connectionsin which lower functional connectivity (i.e., reduced positive correlations and/or increased anticorrelations) was associated with higher intelligence. Edges are weighted by level of correlation reported in the original studies. In the case where no *r-*value was provided (i.e., in between-group contrasts) edges were weighted at the minimum value for visualization purposes (±0.25).

Results from our mapping of studies (Table 1) assessing the relationship between resting state functional connectivity and intelligence scores are presented in Fig. 1. Significant patterns of pairwise functional connectivity positively associated with intelligence (Fig. 1a) suggest a key role for connections between prefrontal and frontal cortices comprising the dorsal attention network (dark green). Significant brain-behaviour associations were also observed for the posterior cingulate/precuneus (red, default-mode network), the superior parietal cortex (yellow, fronto-parietal network) and the occipital cortices (dark blue, visual network). Resting state functional connectivity between bilateral prefrontal cortices encompassing the dorsal attention network and the right insula (salience network, black) was also associated with intelligence scores.

Correlations between lower resting state functional connectivity (i.e., reduced positive correlations and/or increased anticorrelations) and higher intelligence scores (Fig. 1b) involved connections within cortical areas comprising the default-mode network (red) as well as functional interactions between these areas and regions within the dorsal attention network, including the visual, cingulo-parietal and somatosensory (both hand and mouth) regions.

### Empirical analysis of resting state functional networks supporting human intelligence using HCP data

Though suggestive, the above results represent merely the overlap of findings from a small number of studies with varying sample sizes and regions of interest. Thus, to empirically test the hypothesis that resting state connectivity correlates of intelligence extend beyond the fronto-parietal network, we utilized resting state data from the Human Connectome Project. Specifically, we assessed positive and negative linear relationships between whole-brain resting state functional connectivity and intelligence scores of 317 unrelated participants (i.e., participants did not have the same mother or father).

**Figure 2:**
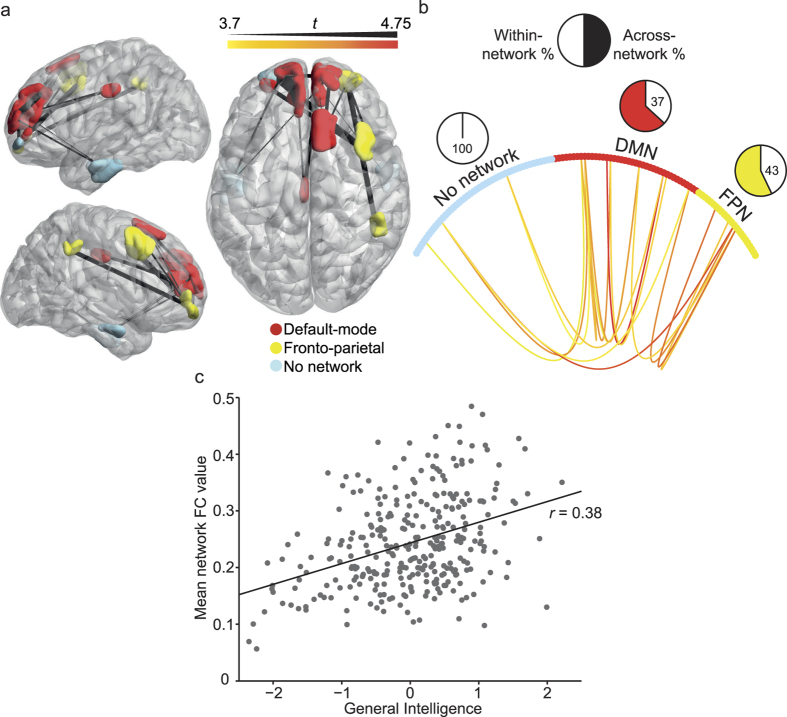
Network-intelligence analysis on 317 independent HCP participants. (**a**) Pairwise functional connections associated with intelligence scores [*p* = 0.045 (extent), *p* = 0.032 (intensity), both FWE corrected at the network level]. Cortical colours reflect their network allegiance, and edge weights reflect the uncorrected edge t-statistics. Note that the light blue regions in (**a**) were not linked to a specific network by Gordon and colleagues^25^. (Panel b) shows the same results as those depicted in (panel a), but outside of anatomical space. Here the edge t-statistics are represented by colour. Circles represent network nodes comprising the default-mode and fronto-parietal and non-affiliated networks. Pie charts show the percentage of significant connections that were within (white) or across (coloured) different networks. (**c**) Scatterplot of the average functional connectivity (FC) values in the whole implicated network (panel a) as a function of general intelligence scores (r = 0.38), DMN = default-mode network, FPN = fronto-parietal network.

Functional connectivity in a concentrated resting state network comprising regions of the fronto-parietal (yellow), default-mode (red), and cortex not associated with any specific network (aqua; note that these regions have been labeled as default-mode in other parcellations^26^) showed a significant positive relationship with intelligence scores (p = 0.045/0.032 familywise error corrected at network level for extent and intensity effects, respectively; see Methods for details, Fig. 2). Specifically, the resulting regions included the bilateral superior medial frontal cortex, superior orbital gyrus and temporal cortex, as well as the left middle cingulate cortex and right middle frontal and supramarginal gyrus (details in Table 2). No other networks were implicated in the analysis. Connections within the default-mode and fronto-parietal networks accounted for the majority of edges detected (Fig. 2b). A follow-up correlation between the mean connectivity value of all implicated edges and general intelligence (as measured by the average of z-scored fluid and crystallized intelligence measures) showed that higher *positive* connectivity values were associated with higher intelligence scores (r = 0.38).

No associations were found between increased intelligence and decreased resting state functional connectivity. Considering the large sample size this is unlikely to be related to a lack of statistical power. Nevertheless, we performed the NBS again with two lower exploratory statistical thresholds (*t* = 3.0 and *t* = 2.5). No networks showed significant negative associations between intelligence and functional connectivity using these lower statistical thresholds.

## Discussion

We assessed whether the dominant parieto-frontal integration theory of intelligence (P-FIT)^9^ can be extended to networks supporting intelligence in task-free contexts (i.e., at rest) by conducting a functional connectivity analysis of Human Connectome Project data. Specifically, we tested whether resting state functional connectivity within frontal and parietal brain regions, and between these regions and the rest of the brain, can account for individual variability in intelligence scores. While our findings confirm a key role for fronto-parietal networks in supporting intelligence, they also highlight the importance of connectivity between regions associated with the fronto-parietal, default-mode and regions not strongly associated with homogeneous networks (although these regions have been identified as comprising the default-mode network before^26^), particularly in the prefrontal cortex. More broadly, our results suggest that interactions between fronto-parietal and default-mode networks are important for explaining individual differences in intelligence in a state of rest.

Recent evidence suggests that the default-mode and frontal-parietal networks represent overarching systems of the brain, composed of several sub-networks that dynamically interact^27^,^28^. Engaging in demanding external tasks has traditionally been associated with increased activity and functional connectivity in fronto-parietal networks, on the one hand, and reduced activity and connectivity in default-mode areas on the other^29^. The opposite functional relationship between these two systems has also been observed during the resting state^30^,^31^. Likewise, during cognitive tasks, it has been shown that individuals with higher and lower intelligence tend to activate these networks differentially^32^,^33^. Specifically, individuals with higher intelligence deactivate the default-mode network less (i.e., a smaller task-induced decrease in the BOLD signal^32^) and activate fronto-parietal and cingulo-opercular network regions more than individuals with lower intelligence^16^,^17^,^32^. Evidence for the second claim, however, is mixed^34^,^35^.

Somewhat at odds with this functional dichotomy between fronto-parietal and default-mode network activity, our analyses suggest that greater *cooperation* (i.e., greater positive correlations) between distinct brain regions comprising default-mode and fronto-parietal networks in the resting state are associated with higher intelligence scores. This pattern of connectivity-intelligence associations is consistent with other findings suggesting that higher global network efficiency is related to higher general intelligence measures^14^ and that the across-network connectivity of the fronto-parietal network is critical for fluid intelligence^36^. More broadly, our findings are compatible with recent conceptualisations of default-mode network function as critical in maintaining a large “dynamic repertoire” of possible neural states at rest^37^,^38^, facilitating the flexible emergence of task-specific dynamics^39^,^40^,^41^.

How the brain self-reorganizes to achieve optimal configurations of functional networks across individuals with varying levels of intelligence is an open question. Recent neuroimaging work has suggested that transient cooperation between different neural systems, including fronto-parietal, cingulo-opercular and default-mode networks, is integral to complex cognitive tasks such as reasoning^42^,^43^, memory recollection^44^ and working memory performance^39^,^45^. Future studies should test the notion that individual differences in intelligence rely on dynamic, context-specific, reconfigurations of local activity and connectivity within a diffuse system comprising fronto-parietal, cingulo-opercular and default-mode regions^46^.

A strength of the current work is the use of a statistically robust network-based method to isolate brain-intelligence associations at rest. While sensitive to network-level associations between functional connectivity and intelligence, our approach may have overlooked edge-specific associations detected in previous work (see Fig. 1). For example, we found no significant negative association between differences in individual functional connectivity strength and intelligence, despite two previous studies included in our explorative mapping reporting such a relationship^10^,^24^. To enhance comparability, we attempted to keep the current analysis as similar to previous work as possible. In fact, both our study and previous work used similar data for calculating functional connectivity (i.e., Z-normalised pairwise Pearson correlations on data without global signal regression). One possible explanation for the discrepancy between our work and earlier results may relate to the use of different statistical methods to infer connectivity-intelligence associations. Previous work used edge-specific correlations with intelligence scores. In contrast, our analysis focused upon significant relationships at the level of whole brain networks. It is possible our approach was less sensitive to circumscribed negative associations between functional connectivity and intelligence. Finally, while our study assessed the functional relationship between pairwise changes in connectivity and intelligence, other studies assessed the link between intelligence and more complex measures of functional connectivity patterns (see Supplementary Table 2). However, we note that these analyses broadly validated the current results by implicating key default-mode regions and other brain areas (see Supplementary Fig. 1).

In summary, our study provides a novel characterization of large-scale networks that explain individual differences in intelligence in a state of rest. Our results suggest that intelligence is supported by activity within a diffuse neural system comprised of brain regions encompassing fronto-parietal and default-mode networks. Consistent with these findings, we propose the influential parieto-frontal intelligence theory (P-FIT) may need to be extended to address context-specific network interactions. The functional links between transitions from diffuse resting state dynamics and more segregated task dynamics and intelligence will be an important topic for ongoing research.

## Methods

### Explorative mapping of relationship between intelligence and resting state networks

For the explorative mapping of data on the relationship between intelligence scores and intrinsic neural activity we performed a manual literature search of English-language peer-reviewed fMRI studies linking measures of pairwise resting state functional connectivity with behavioural measures of intelligence in healthy human adults (Table 1). The literature review was conducted using PubMed, Web of Science^®^ (Thomson Reuter) and Scopus^®^ (Elsevier), and was last updated the 9^th^ of December 2015. Corresponding authors were contacted and asked to provide additional details or whole-brain results if these were not included in the published papers. Our final sample included data from 207 healthy adult participants. In one case^10^,^12^, the same participant cohort was used across two studies. However, in these studies orthogonal region-of-interest analyses were conducted. Conversely, a recent study was not included^47^ because it involved the same cohort and similar analyses as an already included study^24^. Note that we included studies that utilised both individual differences and group differences in intelligence (details in Table 1). We also conducted a summary of several studies that investigated global and local changes in functional connectivity (Supplementary Fig. 1 and Table 2). Due to the lack of overlap in analysis methods, the outcome was not included in the final analysis.

Cortical regions resulting from the above mapping were transposed into a common functional brain parcellation comprising 333 cortical regions^25^. This brain parcellation was selected because it has been shown to be a more refined, homogeneous extension of widely used, functionally defined resting state parcellations^26^,^48^. It is important to note that some of the networks isolated in the adopted parcellation represent sub-networks of the fronto-parietal network defined by the P-FIT. Specifically, the fronto-parietal, cingulo-opercular/parietal and dorsal/ventral attention networks defined by the current brain parcellation are considered to be part of the same fronto-parietal system in the P-FIT. Each region was then mapped into the adopted parcellation by generating 5 mm radius spheres from the reported MNI coordinates and quantitatively assessing the spatial overlap with regions of the adopted parcellation. A parcel was defined as overlapping with the region(s) reported in a previous study when it covered at least 20 contiguous voxels (1 mm^3^) of the MNI sphere. In instances where several parcels were implicated from a single coordinate, only the parcel that overlapped the most was included. Edges were drawn between implicated parcels using their associated *r*-values from the original studies. Note that changing our criteria for an overlapping region, either by increasing or decreasing the voxel limit, or by increasing the sphere size to 10 mm, yielded very similar results to those reported below.

### Analysis of intrinsic functional networks supporting human intelligence using HCP data

We next conducted an analysis of data from a large, independent sample of healthy adult participants, to examine associations between intelligence and functional connectivity across the whole brain in a task-free context. The relationship between measures of intelligence and neural activity was assessed using high quality resting state fMRI data from the HCP^15^. Specifically, we used data from 317 genetically unrelated participants included in the S900 data release (173 female, M_age_ = 28.43 years, SD_age_ = 3.77, range_age_ = 22–36 years). Two relevant behavioural tasks were used as measures of general intelligence. The Penn’s Progressive Matrices (PMAT), a shortened version of Raven’s Progressive Matrices^49^, was used as a measure of fluid intelligence (M = 17.06, SD = 4.77, range = 4–24). In the Raven’s Progressive Matrices participants are presented with puzzles containing visual patterns with a piece missing. They are instructed to ‘fill in the blank piece’ from a given selection of possible answers. The Picture Vocabulary Test, a component of the National Institutes of Health toolbox, was adopted as a measure of crystallized intelligence^50^ (M = 116.59, SD = 9.57, range = 92.84–153.09). In this task participants are presented with an audio recording of a word, and are shown four pictures. They are asked to select the picture that most closely matches the meaning of the spoken word. Individual scores on the PMAT and Picture Vocabulary Test were significantly correlated (*r* = 0.38, *p* < 0.0001).

#### HCP Data Preprocessing and Analysis

Data consisted of whole brain echo-planar images (EPIs) with sub-second temporal resolution (time repetition of 720 ms) and high spatial resolution (2 mm^3^ voxels)^51^,^52^. The data used for this study were downloaded as per the Human Connectome Project minimally preprocessed pipeline with denoising procedures (for details see^53^,^54^) and included both left-to-right and right-to-left acquisitions from the first resting state dataset (i.e., resting state fMRI 1 FIX-denoised package). The average time series from the voxels comprising each of the 333 regions in the adopted parcellation^25^ were extracted using the Matlab toolbox DPARSF V.3^55^. As per the studies included in the resting state explorative mapping^10^,^12^,^23^,^24^, we calculated functional connectivity per participant in each acquisition as a Pearson correlation between each pair of regions, which were subsequently Fisher-Z transformed. Each pair of Z-matrices (left-to right, and right-to-left) was then averaged resulting in a 333 × 333 functional connectivity matrix for each of the 317 participants. Note that no global signal regression was performed for consistency with previously published studies included in the resting state explorative mapping.

To assess the relationship between resting state functional connectivity and individual intelligence scores we used the network based statistic (NBS^56^,^57^, https://sites.google.com/site/bctnet/comparison/nbs). The NBS is a powerful and sensitive statistical tool that controls for Type I error at the network-level. The use of NBS represents a distinct advantage in term of sensitivity over previous studies that corrected for multiple comparisons at the edge level^10^,^23^. Unthresholded functional connectivity matrices were first used as input into the NBS^57^. All possible pairs of connections (333 × 332/2 = 55,278) were examined for putative associations with intelligence. To this end, Z-normalised fluid intelligence scores (PMAT) and crystalized intelligence scores (Picture Vocabulary Test) were used as separate variables of interest in the NBS. Age and gender were considered as covariates of no interest. Following this procedure, a matrix of brain-behaviour associations was obtained. The matrix was thresholded using an exploratory t-statistic of 3.5. A slightly higher final threshold (t = 3.7) was adopted because it allowed the detection of medium sized effects while discarding small or spurious effects^57^. Note that additional exploratory analyses showed that networks arising using higher or lower t-thresholds resembled the original results. Familywise error corrected (FWE) p-values were ascribed to the resulting networks using a null distribution obtained by 5000 permutations. Only components that survived a network-level threshold of p < 0.05 FWE were declared significant. Analyses were performed using both the extent criterion (number of connections in a network) and intensity criterion (sum of test statistic values in a network) in NBS, for both positive and negative associations with intelligence. It is important to note that the NBS is a network-sensitive method, and does not test for significance at the level of individual edges. Therefore, our analysis provides a network-level characterization of resting state functional connectivity correlates of intelligence that can guide further, and more local, investigations.

Figures were generated using BrainNet Viewer^58^, NeuroMArVL, (http://immersive.erc.monash.edu.au/neuromarvl/) and in-house Matlab scripts.

## Additional Information

**How to cite this article**: Hearne, L. J. *et al.* Functional brain networks related to individual differences in human intelligence at rest. *Sci. Rep.* **6**, 32328; doi: 10.1038/srep32328 (2016).

## Supplementary Material

Supplementary Information

## Figures and Tables

**Table 1:** Characteristics of studies included in the resting state explorative mapping.

Author	Sample			Behavioural measure	Brain-behaviour relationship	Analysis type	Regions of interest
	N	Males	Age (M ± SD)				
Song *et al.*	59^a^	49%	24.6 ± 3.5	WAIS (Chinese)	Correlation	Seed to voxel-wise whole-brain analysis	Bilateral DLPFC
Song *et al.*	59^a^	49%	24.6 ± 3.5	WAIS (Chinese)	Correlation	Multi-region pairwise analysis	13 default-mode regions defined by seeding the PCC
Pamplona *et al.*	29	52%	26.8 ± 5.8	WAIS (Portuguese-Brazil)	Correlation	Multi-region pairwise analysis.	82 AAL atlas regions^b^
Santarnecchi *et al.*	119	50%	33 ± 13	WASI	Between-group: high and low comparison defined by median split	Seed to voxel-wise whole-brain analysis	Six seed regions defined by prior VHMC analysis

Note:

^a^The samples used in the indicated studies were not independent.

^b^MNI centroids were used as regions of interest. WAIS = Wechsler Adult Intelligence Scale, WASI = Wechsler Abbreviated Scale of Intelligence, DLPFC = dorsolateral prefrontal cortex, PCC = posterior cingulate cortex, AAL = Automated Anatomical Labeling, VHMC = voxel-mirrored homotopic connectivity.

**Table 2:** Regions implicated in the analysis of the Human Connectome Project data.

Gordon region	MNI Coordinates			Resting-state network	Anatomy
	x	y	z		
25	−5.6	42.2	35.1	Default-mode	Superior medial frontal gyrus
26	−1.7	−17.7	39.1	Default-mode	Middle cingulate cortex
114	−27.5	53.6	0	Default-mode	Superior frontal gyrus
115	−23.4	61	−6.8	None	Superior orbital gyrus
128	−53.2	−13	−29.2	None	Inferior temporal gyrus
150	−6.5	54.7	18.1	Default-mode	Superior medial frontal gyrus
151	−15.7	64.7	13.7	Default-mode	Superior frontal gyrus
165	11.9	21.9	59.9	Default-mode	Posterior medial frontal gyrus
167	47.9	−42.5	41.5	Fronto-parietal	Supramarginal gyrus
277	28.4	57	−5.1	Fronto-parietal	Superior orbital gyrus
291	54.7	−7.8	−26.9	None	Inferior temporal gyrus
321	16	61	19.8	Default-mode	Superior medial frontal gyrus
322	8.2	53.8	14	Default-mode	Superior medial frontal gyrus
327	42.4	19.5	48.2	Fronto-parietal	Middle frontal gyrus
328	38.9	9.6	42.7	Fronto-parietal	Middle frontal gyrus

Note: In some cases the implicated parcels cross multiple anatomical boundaries, here we have simply tried to provide the most accurate anatomical description.
