# Supplementary material for: Functional brain networks related to individual differences in human intelligence at rest
Source: Sci Rep. 2016 Aug 26;6:32328. doi: 10.1038/srep32328 (PMC4999800; doi:10.1038/srep32328)
Supplement: Supplementary Information [file srep32328-s1.pdf]

Supplementary materials

Functional brain networks related to individual differences in human intelligence at rest

Luke J. Hearne[1*], Jason B. Mattingley[1,2], Luca Cocchi[1,3]

[1] Queensland Brain Institute, The University of Queensland, Brisbane, Australia.
[2] School of Psychology, The University of Queensland, Brisbane, Australia.
[3] QIMR Berghofer Medical Research Institute, Brisbane, Australia

Corresponding Author: * l.hearne@uq.edu.au

Supplementary Table 1. Characteristics of studies utilizing complex graph metrics included in the supplementary resting-state meta-analysis.

	Sample						
Author	N	Males	Age (M±SD)	Behavioural measure	Brain-behaviour relationship	Analysis type	Regions of interest
Van den Heuval et al.	19	74%	29±7.8	WAIS	Correlation	Global efficiency	Whole brain
Wang et al.	59	49%	24.6±3.5	WAIS (Chinese)	Correlation	Regional Homogeneity	Whole brain
Cole et al.	94	42%	22±4.7	RAPM and CCFT	Correlation	Global brain connectivity	LPFC
Yuan et al.	284	46%	22.8±2.4	RPM	Correlation	Regional Homogeneity	Whole brain
Santarnecchi et al.	98	50%	34±14	WASI	Between-group: high, average and low defined by clustering analysis	Global efficiency	90 AAL atlas regions[a]
Pamplona et al.	29	52%	26.8±5.8	WAIS (Portuguese-Brazil)	Correlation	Local efficiency	82 AAL atlas regions[a]

Note: [a]MNI centroids were used as regions of interest in the case of the AAL template. CCFT = Cattell Culture Fair Test, RPM = Ravens Standard Progressive Matrices, RAPM = Ravens Advanced Progressive Matrices, WAIS = Wechsler adult intelligence scale, WASI = Wechsler abbreviated scale of intelligence, LPFC = lateral prefrontal cortex, AAL = Automated Anatomical Labeling.

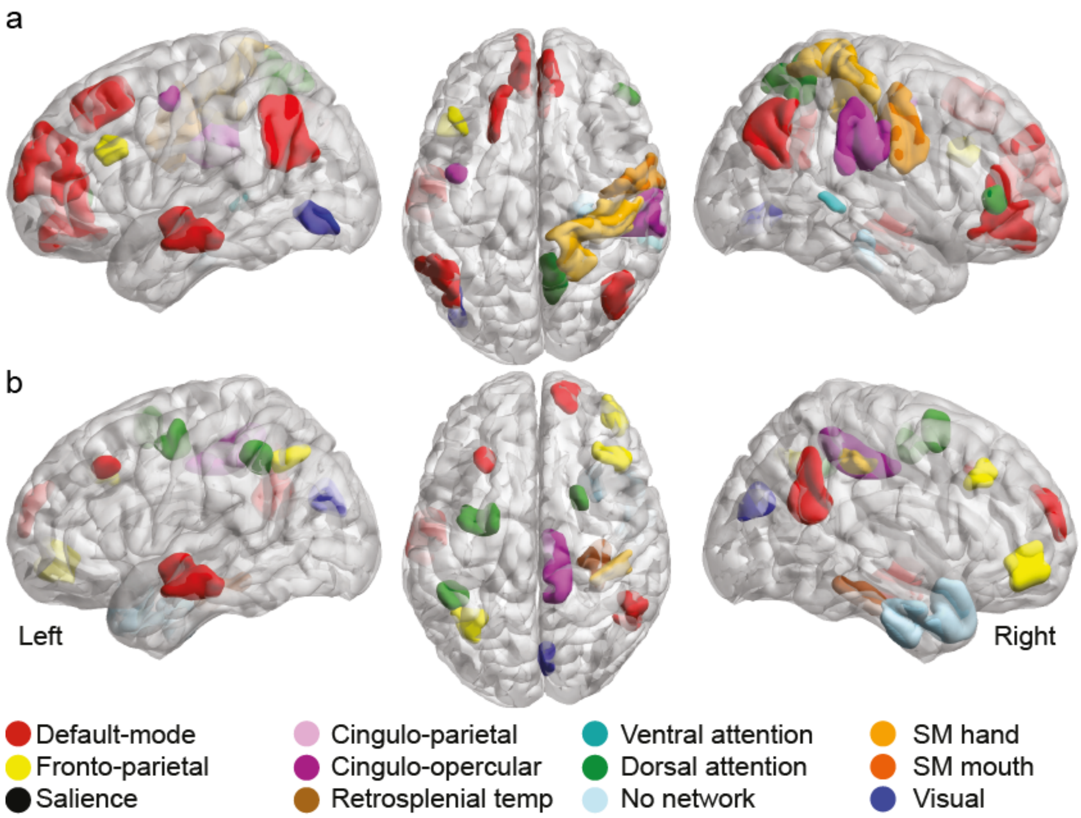

Supplementary Figure 1. Regions of the brain that demonstrated global or local graph properties at rest that were associated with intelligence. a. Efficiency metrics b. Regional homogeneity.
